# *Plasmodium falciparum* Chloroquine-*pfcrt* Resistant Haplotypes in Brazilian Endemic Areas Four Decades after CQ Withdrawn

**DOI:** 10.3390/pathogens12050731

**Published:** 2023-05-17

**Authors:** Rebecca de Abreu-Fernandes, Natália Ketrin Almeida-de-Oliveira, Bianca Ervatti Gama, Larissa Rodrigues Gomes, Aline Rosa De Lavigne Mello, Lucas Tavares de Queiroz, Jacqueline de Aguiar Barros, Maria das Graças Costa Alecrim, Rodrigo Medeiros de Souza, Lilian Rose Pratt-Riccio, Patrícia Brasil, Cláudio Tadeu Daniel-Ribeiro, Maria de Fátima Ferreira-da-Cruz

**Affiliations:** 1 Laboratório de Pesquisa em Malária, Instituto Oswaldo Cruz, Fundação Oswaldo Cruz (Fiocruz), Rio de Janeiro 21041-361, Brazil; 2 Centro de Pesquisa, Diagnóstico e Treinamento em Malária (CPD-Mal), Reference Laboratory for Malaria in the Extra-Amazonian Region for the Brazilian Ministry of Health, Secretaria de Vigilância Sanitária & Fiocruz, Rio de Janeiro 21041-361, Brazil; 3 Centro de Transplante de Medula Óssea Laboratório de Oncovirologia, Instituto Nacional do Câncer, Rio de Janeiro 20230-130, Brazil; 4 Laboratório de Bioquímica e Proteínas de Peptídeos, CDTS Centro de Desenvolvimento Tecnológico em Saúde, Fiocruz, Rio de Janeiro 21041-361, Brazil; 5 Núcleo de Controle da Malária/Departamento de Vigilância Epidemiológica/Coordenação Geral de Vigilância em Saúde/SESAU-RR, Boa Vista 69305-080, Brazil; 6 Fundação Instituto de Medicina Tropical Dr Heitor Vieira Dourado (FMT-HVD), Amazonas 69040-000, Brazil; 7 Centro de Pesquisa em Doenças Infecciosas, Universidade Federal do Acre, Rio Branco 69920-900, Brazil; 8 Instituto Nacional de Infectologia Evandro Chagas, Fiocruz, Rio de Janeiro 21040-361, Brazil

**Keywords:** chloroquine, chemoresistance, malaria, *P. falciparum*, *pfcrt*

## Abstract

(1) Background: Malaria is a public health problem worldwide. Despite global efforts to control it, antimalarial drug resistance remains a great challenge. In 2009, our team identified, for the first time in Brazil, chloroquine (CQ)-susceptible *Plasmodium falciparum* parasites in isolates from the Brazilian Amazon. The present study extends those observations to include survey samples from 2010 to 2018 from the Amazonas and Acre states for the purpose of tracking *pfcrt* molecular changes in *P. falciparum* parasites. (2) Objective: to investigate SNPs in the *P. falciparum* gene associated with chemoresistance to CQ (*pfcrt)***.** (3) Methods: Sixty-six *P. falciparum* samples from the Amazonas and Acre states were collected from 2010 to 2018 in patients diagnosed at the Reference Research Center for Treatment and Diagnosis of Malaria (CPD-Mal/Fiocruz), FMT-HVD and Acre Health Units. These samples were subjected to PCR and DNA Sanger sequencing to identify mutations in *pfcrt* (C72**S**, M74**I**, N75**E**, and K76**T***).* (4) Results: Of the 66 *P. falciparum* samples genotyped for *pfcrt*, 94% carried CQ-resistant genotypes and only 4 showed a CQ *pfcrt* sensitive-wild type genotype, i.e., 1 from Barcelos and 3 from Manaus. (5) Conclusion: CQ-resistant *P. falciparum* populations are fixed, and thus, CQ cannot be reintroduced in malaria falciparum therapy.

## 1. Introduction

Tens of thousands of years after the *Plasmodia* that infected hominids became established as parasites that cause disease in humans, malaria is still a major public health problem worldwide in the third millennium of the Christian era. According to the World Malaria Report, 247 million cases and 619 thousand malaria-related deaths were reported in 2021 [1]. *Plasmodium falciparum* is responsible for the most virulent and dangerous malaria in humans [1,2]. In 2021, in the Brazilian Amazon Basin, 138,988 cases—representing 99.92% of the Brazilian cases—were reported. Among them, 21,614 (15.55%) were caused by *P. falciparum* [2].

Despite numerous advances in the use and efficacy of vaccines, there is still heavy reliance on antimalarials for the prevention and treatment of malaria; these drugs are considered the most important malaria control measures [3]. However, with the continued use of antimalarials, *P. falciparum* gradually develops drug resistance that spreads rapidly [4]. Therefore, antimalarial drug resistance has become one of the major challenges in eliminating the disease [5]. The emergence of drug-resistant strains may be influenced by parasites and host factors, such as parasite mutation frequency, patient adherence to therapy, selection pressure, and host immunity, in addition to drug quality [6].

Before artemisinin-based combination therapies (ACTs) were approved worldwide as first-line therapy for uncomplicated falciparum malaria in 2007, chloroquine (CQ) was widely used in Brazil, especially up to the 1980s, to treat acute infections with *P. falciparum* as a safe, inexpensive, and effective antimalarial drug [7,8,9]. Mefloquine was then introduced as a therapeutic alternative for multidrug-resistant falciparum malaria; it was used until the introduction of ACTs in Brazil, with relative safety, alone or in association with artemisinin derivatives in cases of severe malaria and multidrug-resistant *P. falciparum* parasites [10]. Currently, after reports of cases of resistance to mefloquine [10], this drug is only used in combination with artesunate for the treatment of acute, uncomplicated malaria caused by *P. falciparum*. It is indicated for cases of *P. falciparum* mono-infection, as well as for mixed infections with *P. vivax* (with subsequent treatment of its hypnozoite forms).

Since the first reports of *P. falciparum* resistance to antimalarial drugs in the nineteenth century, molecular epidemiological surveillance has been essential for the early detection and prevention of the spread of resistant parasites [11,12] by identifying and monitoring genetic polymorphisms associated with parasite resistance, mainly single nucleotide polymorphisms (SNPs) [8,12].

Mutations in the *P. falciparum* chloroquine resistance transporter gene (*pfcrt*), a member of the drug metabolite transporter superfamily, have been associated with reduced susceptibility to CQ [11]. The K76**T** *pfcrt* polymorphism is considered the molecular marker of CQ resistance (CQR) [13] and is associated with CQ treatment failure [14,15]. However, studies have suggested that the K76**T** mutation does not act alone but in conjunction with other *pfcrt* mutations, such as those at positions 72, 73, 74, and 75 [16,17,18]. Thus, CQR strains of *P. falciparum* could carry triple CV**IET** (mostly in Southeast Asia and Africa) or double **S**VMN**T** mutants (South America) [19,20,21,22].

In 2009, our team identified, for the first time in Brazil, the presence of *P. falciparum* parasites sensitive to CQ in the Brazilian Amazon [23]. The present study extends these observations to include survey samples from 2010 to 2018 from the Amazonas and Acre. Due to the limitations of in vivo and in vitro studies to survey chemoresistant parasites in endemic areas where reinfections are common, molecular analysis of parasite mutations associated with chemoresistance is an important tool. These findings prompted us to conduct a study to track molecular changes in *P. falciparum* parasites through the investigation of SNPs in the *pfcrt* gene in parasites from the Amazonas and Acre Brazilian states.

## 2. Materials and Methods

### 2.1. Blood Samples and Malaria Diagnosis

**Figure 1:**
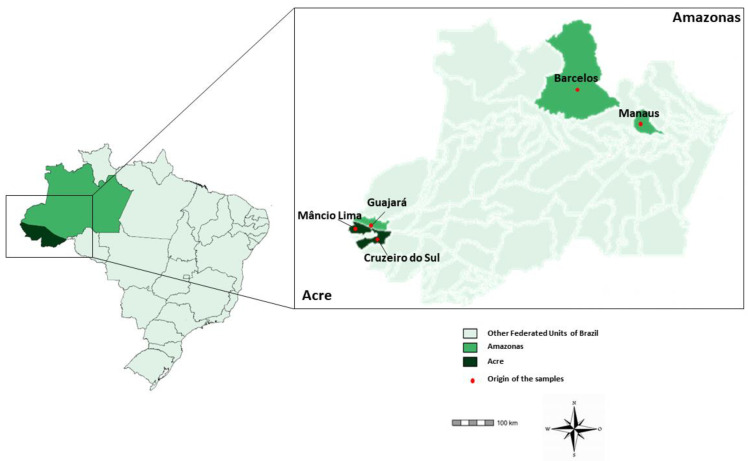
Brazilian map highlighting the Acre and Amazonas states and the municipalities of parasite infection.

Samples were collected from *P. falciparum*-infected symptomatic patients who attended the Ambulatório de Síndromes Febris Agudas/Acute Febrile Syndrome Outpatient Clinic at the National Institute of Infectology (INI), Rio de Janeiro, a member of the Reference Center for Research, Diagnosis, and Training of Malaria—CPD-Mal/Fiocruz, RJ for the Extra-Amazonian region (22° 54′ S W 43° 12′ W). Blood samples were also collected in Manaus (3.1190° S, 60.0217° W), the capital of Amazon state, at the Fundação de Medicina Tropical Doutor Heitor Vieira Dourado (FMT-HVD) and in field conditions in the municipality of Guajará (bordering the Amazonas and Acre states; 02°58′18′′ S and 57°40′38′′ W) and in two municipalities of Acre state: Cruzeiro do Sul (07°37′50′′ S and 72°40′13′′ W) and Mâncio Lima (07°36′49′′ S and 72°53′47′′ W) (Table 1 and Figure 1). Independently of blood collection locality, *P. falciparum* diagnosis was made by light microscopy (Giemsa-stained thick blood droplets) in situ and by species-specific polymerase chain reaction (PCR) [24] at the Fiocruz Malaria Research Laboratory—the headquarters of the CPD-Mal—where the samples were stored.

### 2.2. DNA Extraction, Amplification, and Sequencing

The DNA from 1 mL blood samples was extracted using a QIAamp™ DNA Blood Midi Kit (QIAGEN), according to the manufacturer’s instructions. PCRs were performed to amplify the *pfcrt* fragment gene according to previously described protocols [25]. PCR products were analyzed by electrophoresis on 2% agarose gel and visualized under a UV transilluminator (DigiDoc-It, UVP, Upland, CA, USA). Each PCR product was purified using Wizard™ SV Gel and the PCR Clean-Up System (Promega, WI, USA), following the manufacturer’s procedure. Purified DNA sequencing was carried out through Big Dye™ Terminator Cycle Sequencing Ready Reaction version 3.1 (Applied Biosystems, Carlsbad, CA, USA), with 3.2 μM of forward and reverse PCR primers. DNA sequences to investigate C72**S**, M74**I**, N75**E/D**, and K76**T** were determined using an ABI Prism DNA Analyzer™ 3730 (Applied Biosystems, CA, USA), on the Fiocruz Genomic Platform PDTIS/Fiocruz RPT01A. Nucleotide sequences were aligned using the ClustalW multiple sequence aligner in the BioEdit software [26]. The PF3D7_1343700 strain was used as a reference sequence (from PlasmoDB: http://www.plasmoDB.org, accessed on 20 March 2023). DNA sequences were deposited in GenBank (the NIH’s genetic sequence database; www.ncbi/nlm/nih.gov/GenBank, accessed on 21 March 2023) with the accession numbers OQ672386-OQ672451.

## 3. Results

PCR amplicons (145-bp) of the *pfcrt* gene covering codons 72–76 were sequenced. The prevalence of C72**S** and K76**T** mutations was 92% (61/66). All parasites from Cruzeiro do Sul and Mâncio Lima Acre municipalities, as well as those from Guajará Amazonas municipality, showed both C72**S** and K76**T** polymorphisms, i.e., exhibiting the double mutant **S**VMN**T** haplotype. Only four samples from Amazonas municipalities—three from Manaus and one from Barcelos—were *pfcrt* CVMNK wild type. The remaining sample from Manaus presented mutations at codons 74 (M74**I**), 75 (N75**E**), and 76 (K76**T**), displaying the triple mutant CV**IET** haplotype (Table 2).

## 4. Discussion

The *P. falciparum* has demonstrated its ability to develop resistance to all drugs that have been used against it on a large scale, continuously threatening global efforts to control malaria, a leading infectious cause of human morbidity and mortality. Although Africa bears by far the heaviest burden of malaria, CQ-resistant parasites first emerged in Southeast Asia and America [27]. This fact underscores the importance of studying and understanding the genotype of circulating parasites in malaria-endemic areas, since the strong pressure of drugs can lead to the establishment of drug resistance alleles, even if they generate a fitness cost for parasites in the absence of drug pressure [28]. Therefore, understanding the evolution of drug target genes under changing drug policy is crucial for drug efficacy monitoring using molecular markers.

Polymorphisms in the amino acid positions 72–76 of the *pfcrt* gene are reliable markers for CQR of *P. falciparum* parasites, of which K76**T** mutation is predominant [16,29]. In our study, the high prevalence of the 76**T** allele in isolates from Acre and Amazonas agrees with other studies in Brazil [30,31], and 76**T** mutation was found in two CQR haplotypes, CV**IET** and **S**VMN**T**, which was consistent with our initial hypothesis.

The CV**IET** haplotype is predominant in many African [21,32] and Southeast Asian countries in which CQ has been withdrawn for at least ten years after [33,34,35]; however, it has also been observed in *P. falciparum* parasites from South America [36], while **S**VMN**T** is dominant in South America and Oceania [37]. In Brazil, the CV**IET** haplotype was rarely encountered (it was found in only one sample from the municipality of Manaus), as previously seen in isolates from Amazonas and Rondônia [38]. This low CV**IET** haplotype frequency in Brazilian isolates suggests that this allele might have been recently introduced through human migration between Africa and South America.

On the other hand**,** the **S**VMN**T** haplotype is mainly detected in South America and is rarely found in Africa [21,39] and Southeast Asia [33,34,35]. A study released in 2022 claimed that the **S**VMN**T** haplotype originated independently in South America [22], and it was suggested that this haplotype might be responsible for the initial CQR sweeps across the Amazon in the early 1960s [38]. **S**VMN**T** was more prevalent than other mutant haplotypes found in this survey, corroborating previous findings in Brazil [24] and showing its persistence, despite a decline in CQ use.

Our team reported, for the first time, the presence of wild-type haplotypes circulating in Brazilian isolates [23]. Now, almost ten years later, we found this haplotype in only four isolates from the Amazonas state (three from Manaus and one from Barcelos). Considering that in Brazil, CQ has not been used to treat *P. falciparum* since the 1980s, a higher percentage of parasites sensitive to CQ would theoretically be expected. In fact, up to 90% of the samples showing a reversal of *pfcrt* from the CQ-resistant to the CQ-sensitive genotype were taken 19 years after the withdrawal of CQ in Kenya, in contrast to the results observed in the present study [40]. Thus, the high level of K76**T** *pfcrt* mutations observed in Brazilian endemic areas is suggestive of a sustained CQ pressure on *P. falciparum* parasites. In fact, CQ is used in the treatment of vivax malaria, leading to continuous exposure to this drug. Alternatively, the presence of a K76**T** mutation might have a positive effect on the fitness of the parasite, settling down in the parasitic population of the region, or lesser opportunities for competition because of a lower rate of polyclonal infections and a relative lack of competing wild-type parasites [41] Additionally, C350**R** substitution on the *pfcrt* gene could also participate in the restoration of CQ susceptibility, as suggested elsewhere [28]. Since the C350**R** mutation is in exon 10, and the primers we used flank the exon 2 region, comprising amino acids located at codons 43-91, studies are in progress to answer this question.

## 5. Conclusions

We conclude that the *P. falciparum* **S**VMN**T** haplotype is fixed in Brazilian endemic areas. This notwithstanding, molecular surveillance of the *P. falciparum pfcrt* gene to monitor trends in the emergence and spread of CQ-sensitive *P. falciparum* haplotypes in parasites in Brazilian endemic areas can help to understand the evolutionary dynamics of antimalarial drug resistance in the Amazon Basin, where more than 99% of Brazilian malaria cases occur and where *P. falciparum* resistance to CQ keeps being the rule.

## Figures and Tables

**Table 1:** Localities of *P. falciparum* parasite blood collection by year.

Year	Sample Collection				
	Rio de Janeiro (*n* = 2)	Amazonas (*n* = 32)		Acre (*n* = 34)	
	CPDMAL ^1^	FMT-HVD ^2^	GJ ^3^	CZS ^4^	ML ^5^
2010	-	22	-	-	-
2013	-	4	-	-	-
2014	-	1	-	-	-
2016	-	-	1	11	8
2017	1 ^6^	-	-	-	-
2018	1 ^7^	-	2	9	6

^1^ Reference Center for Malaria Treatment and Diagnosis of Brazilian Ministry of Health. ^2^ Fundação de Medicina Tropical Doutor Heitor Vieira Dourado, Amazonas state. ^3^ Guajará municipality, Amazonas state; ^4^ Cruzeiro do Sul municipality, Acre state; ^5^ Mâncio Lima municipality, Acre state; ^6^ Manaus municipality, Amazonas state; ^7^ Barcelos municipality, Amazonas state.

**Table 2:** *pfcrt* haplotypes in 66 *P. falciparum* samples from Amazonas (Manaus, Barcelos, and Guajará/GJ) and Acre (Cruzeiro do Sul/CZS and Mâncio Lima/ML) Brazilian states.

Haplotype ^1^	Locality					Total (%)
	Manaus (*n* = 28)	Barcelos (*n* = 1)	GJ (*n* = 3)	CZS (*n* = 20)	ML (*n* = 14)	
CVMNK ^2^	3 (11%)	1 (100%)	-	-	-	4 (6%)
**S**VMN**T**^3^	24 (86%)	-	3 (100%)	20 (100%)	14 (100%)	61 (92%)
CV**IET** ^4^	1 (4%)	-	-	-	-	1 (1%)

^1^ The bold character represents a non-synonymous mutation. ^2^ Reference Pf3D7 wild haplotype sequence. ^3^ S: codon 72; T: codon 76. ^4^ I: codon 74; E: codon 75; T: codon 76.

## Data Availability

Data supporting the conclusions of this article are included within the article. The datasets used and/or analyzed during the present study are available from the corresponding author on reasonable request.
